# Exploring potential hidden aspects of quantum field theory through numerical solution of the Klein–Gordon equation using the Yee algorithm

**DOI:** 10.1038/s41598-025-24512-8

**Published:** 2025-11-19

**Authors:** Babak Honarbakhsh

**Affiliations:** Faculty of Electrical Engineering, Shahid Beheshti University, P.O. Box 19839 69411, Tehran, Iran

**Keywords:** Klein–Gordon equation, Maxwell–Heaviside equations, Finite‑difference time‑domain method, Yee algorithm, Conserved Maxwellian Fields, Quantum field theory, Mathematics and computing, Physics

## Abstract

This study presents a novel reformulation of the Klein–Gordon (KG) equation by embedding it within a system of first-order Maxwell–Heaviside (MH)-like equations, enabling its numerical solution using the finite-difference time-domain method based on the Yee algorithm. This approach extends the scalar KG field into a pair of fictitious Maxwellian vector fields. This reformulation not only provides an efficient computational framework, capable of handling nonlinearity and inhomogeneity, but also introduces a first-order structure with symmetric field dynamics. Plane-wave quantization of these fields reveals a conserved, non-negative quantity, forming what is termed Conserved Maxwellian Fields (CMFs), that addresses the longstanding issue of negative probability density in the conventional KG theory. Furthermore, the resulting CMFs exhibit deep structural analogies with Dirac spinors, particularly in three spatial dimensions, where only two CMF modes exist with monopole-like divergence. These findings bridge the gap between scalar field dynamics and electromagnetic field theory, offering both computational utility and potential insight into hidden structures in quantum field theory.

## Introduction

The numerical solution of the Klein–Gordon (KG) equation has a long history and remains an active area of research^1–31^. Originally, the KG equation is a scalar, linear, homogeneous, constant-coefficient partial differential equation that is second-order in both space and time^32^. However, by relaxing the linearity constraint and allowing the coefficients to vary spatially, the KG equation can model a variety of physical phenomena, including solitary wave propagation in complex media^15^. In particular, the well-known sine–Gordon equation is a special case of a generalized KG equation^33^.

Among the various numerical techniques, finite-difference (FD) schemes are not only simple to implement but also highly flexible, due to their straightforward handling of material inhomogeneities and nonlinearities. This makes them particularly well-suited for solving the KG equation in complex media. In fact, the application of numerical methods other than FD to the KG equation remains relatively rare^12,19,21^.

Given that the likelihood of spurious solutions often increases with the order of the differential equation, it is natural to consider reformulating the KG equation as a system of first-order equations^34^. Notably, the mathematical form of the KG equation matches that of the inhomogeneous electromagnetic (EM) wave equation in the Lorenz gauge. This observation suggests that the Maxwell–Heaviside (MH) equations are a natural first-order candidate for such reformulation^35^.

Accordingly, the KG equation can be embedded as one of six components within two fictitious Maxwellian vector. This construction is equivalent to extending the scalar KG field into a higher-dimensional Maxwellian field, whose domain and range fully encompass those of the original scalar field. In doing so, a conceptual bridge is established between the KG and MH equations, allowing the extensive numerical framework developed in computational electromagnetics (CEM) to be adapted for efficient solution of the KG equation. In particular, this enables the application of the widely used finite-difference time-domain (FDTD) method—a FD scheme based on the Yee algorithm^36,37^. The Yee Algorithm, introduced in 1966, is a cornerstone FDTD method for the numerical solution of Maxwell’s equations. It is built on a staggered grid in both space and time, often referred to as the Yee grid, where electric and magnetic field components are placed at alternating half-grid points. This ingenious arrangement allows for the derivatives in Maxwell’s equations to be approximated using second-order accurate central differences. The algorithm uses a leapfrog integration scheme, where the electric and magnetic fields are updated in an alternating fashion, staggered by half a time step. This structure makes the method explicit, numerically stable, and energy-conserving, leading to its widespread adoption for analyzing EM wave propagation, scattering, and resonance in complex materials and devices.

The aforesaid formulation introduces five auxiliary scalar fields in addition to the primary KG function. While these extra components currently lack direct physical interpretation, they may suggest unexplored structures or hidden degrees of freedom relevant to quantum field theory (QFT). The present work validates the feasibility of the proposed reformulation by implementing it using the Yee algorithm. Besides, through field quantization based on plane-wave solutions, a conservation law is shown to exist for a non-negative function. This resolves the long-standing issue of negative probability densities in KG. As well, in the plane-wave regime, the resulting Maxwellian fields exhibit complete symmetry, in the sense that both vector fields become non-solenoidal.

Two additional points are worth noting. First, the similarity between the KG and MH equations was recognized long ago by Alexandru Proca, who introduced what are now known as Proca fields to describe the dynamics of massive spin-1 particles^38^. However, the original Proca equations govern the four-potential and are second-order in both space and time, whereas the MH equations are first-order and act directly on the electric and magnetic fields. Furthermore, although the Proca equations can be expressed in terms of field quantities, they necessarily involve both scalar and vector potentials, and thus are not formulated purely in terms of electric and magnetic fields. Second, the term FDTD can, in principle, refer to any time-domain numerical method based on FD approximations, and many such variants have been applied to the KG equation. However, in the context of CEM, FDTD typically refers to a specific class of methods that solve the MH equations using the Yee algorithm for both spatial and temporal discretization, and its application to the KG equation in this manner is reported here for the first time. The rest of the paper organizes as follows: in section two, the most general form of the KG equation is expressed based on couple of curl equations as in the MH equations and sufficient time-domain equations for numerical solution of the KG equation is derived. Additionally, pseudo codes regarding FDTD implementation are included. Section three validates the proposed FDTD discretization to various test problems and concludes the numerical aspect of this paper. Sections four and five are devoted to the theoretical framework. Specifically, section four reformulates the original KG equation as a symmetric representation of all four MH equations. In this context, the concept of Conserved Maxwellian Fields (CMFs) is introduced, and it is shown how the source terms in the curl equations can be utilized to define such fields. Finally, section five derives plane-wave solutions for CMFs in one, two, and three spatial dimensions.

## Representing generalized Klein–Gordon equation by Maxwellian fields

The most general form of the KG equation is:

1 $$\left( {\frac{1}{{c^{2} }}\frac{{\partial^{2} }}{{\partial t^{2} }} - \nabla^{2} } \right)\psi \left( {{\mathbf{r}},t} \right) + \beta \frac{\partial }{\partial t}\psi \left( {{\mathbf{r}},t} \right) + w\left( {{\mathbf{r}},t} \right)g\left[ {\psi \left( {{\mathbf{r}},t} \right)} \right] + f\left( {{\mathbf{r}},t} \right) = 0$$

where $c = 1/\sqrt {\mu \varepsilon }$ is the speed of light in the medium, $\beta$ is a damping (or dissipation) coefficient, $w$ captures inhomogeneities in the medium, $g$ is, in general, a nonlinear function, and $f$ is an external forcing term^15^. Let 3D time-varying vector fields ${{\varvec{\Psi}}}$ and ${{\varvec{\Phi}}}$ satisfy:

2 $$\left\{ {\begin{array}{*{20}l} {\nabla \times {{\varvec{\Psi}}} = - \mu \frac{\partial }{\partial t}{{\varvec{\Phi}}}} \hfill \\ {\nabla \times {{\varvec{\Phi}}} = + \varepsilon \frac{\partial }{\partial t}{{\varvec{\Psi}}} + {\mathbf{X}}_{\psi } } \hfill \\ \end{array} } \right.$$

wherein ${{\varvec{\Phi}}}$ and ${\mathbf{X}}_{\psi }$ can be regarded as the dual of ${{\varvec{\Psi}}}$ and the source term, respectively. As long as numerical solution using the FDTD method is of interest, $\nabla \cdot {{\varvec{\Psi}}}$ and $\nabla \cdot {{\varvec{\Phi}}}$ are left unconstrained, since the Yee algorithm solves Maxwell’s curl equations. Additionally, let one of the Cartesian components of ${{\varvec{\Psi}}}$ coincide with the KG field variable. Noting that the Cartesian components of the vector Laplacian operator are the same as the scalar Laplacian operator, the analogy between the MH and KG equation can be established if ${\mathbf{X}}_{\psi }$ satisfies

3 $$\nabla \nabla \cdot {{\varvec{\Psi}}} + \mu \frac{\partial }{\partial t}{\mathbf{X}}_{\psi } \equiv \left[ {\beta \frac{\partial }{\partial t}\psi + wg\left( \psi \right) + f} \right]{\mathbf{u}}$$

wherein ${\mathbf{u}}$ is a constant vector. Consequently, the vector extension of the KG equation is equivalent to the propagation of an EM wave within a complex medium that has field-dependent sources. Moreover, any general Maxwell solver can be utilized for the numerical solution of the KG equation. It is important to mention that there are auxiliary conditions associated to (2), including initial conditions (ICs). Specifally, let $\psi \left( {{\mathbf{r}},0} \right) = \psi_{1}$ and $\psi_{,t} \left( {{\mathbf{r}},0} \right) = \psi_{2}$. The imposition of the former is straightforward. To impose the later, it can be assumed that $\left. {\nabla \times {{\varvec{\Phi}}}} \right|_{{\left( {{\mathbf{r}},0} \right)}} = 0$, leading to ${\mathbf{X}}_{\psi } \left( {{\mathbf{r}},0} \right) = \psi_{2} {\hat{\mathbf{u}}}$. Consequently, the time-domain equations that need to be discretized are:

4 $$\left\{ {\begin{array}{*{20}l} {\frac{\partial }{\partial t}{{\varvec{\Phi}}} = - \frac{1}{\mu }\nabla \times {{\varvec{\Psi}}}} \hfill \\ {\frac{\partial }{\partial t}{{\varvec{\Psi}}} = + \frac{1}{\varepsilon }\nabla \times {{\varvec{\Phi}}} - \frac{1}{\varepsilon }{\mathbf{X}}_{\psi } } \hfill \\ {\frac{\partial }{\partial t}{\mathbf{X}}_{\psi } = - \frac{1}{\mu }\nabla \nabla \cdot {{\varvec{\Psi}}} + \frac{1}{\mu }\left[ {\beta \frac{\partial }{\partial t}\psi + wg\left( \psi \right) + f} \right]{\mathbf{u}}} \hfill \\ \end{array} } \right.$$

The numerical solution of the KG equation in all dimensions is of particular interest, prompting further study of the specialized forms of Eq. (4). It is essential to highlight that although the FDTD method is easier to implement than other techniques, such as the Finite Element Method (FEM), there are two key factors that can significantly impact the accuracy of the solution if not properly addressed. The first factor is the order of the update equations. Mathematically, the arrangement of the equations in (4) is not important; however, from a numerical standpoint, the ordering is critical for obtaining accurate results. The second factor involves the Yee algorithm, which is based on central differences in both space and time. When represented using arrays, these differences become either forward or backward differences. Given the presence of an additional vector field in (4), the details of discretization are vital. Therefore, for each case, the relevant pseudocode for numerical implementation is provided, wherein it is assumed that $\mu = \varepsilon = w = 1$ and $\beta = 0$, for brevity. Also, in accordance with (4), “f” and “g” denote predefined functions representing nonlinearity and forcing functions.

### One-dimensional case

Let $\frac{\partial }{\partial x} = \frac{\partial }{\partial y} = 0$ and ${{\varvec{\Psi}}}\left( {z,t} \right) = {\hat{\mathbf{x}}}\psi$. Then, ${{\varvec{\Phi}}}\left( {z,t} \right) = {\hat{\mathbf{y}}}\varphi$ and ${\mathbf{X}}_{\psi } \left( {z,t} \right) = {\hat{\mathbf{x}}}\chi$ which simplifies (4) to:

5 $$\left\{ {\begin{array}{*{20}l} {\frac{\partial }{\partial t}\varphi = - \frac{1}{\mu }\frac{\partial }{\partial z}\psi } \hfill \\ {\frac{\partial }{\partial t}\psi = - \frac{1}{\varepsilon }\frac{\partial }{\partial z}\varphi - \frac{1}{\varepsilon }\chi } \hfill \\ {\frac{\partial }{\partial t}\chi = + \frac{1}{\mu }\left[ {\beta \frac{\partial }{\partial t}\psi + w\left( {{\text{z}},t} \right)g\left( \psi \right) + f} \right] } \hfill \\ \end{array} } \right.$$

In analogy with TEM waves, the above-mentioned solution can be regarded as a $T\Psi \Phi$ quantum wave. Especially, (5) becomes the Telegrapher’s equations governing a uniform lossy transmission line if $g = f = 0$^39^. The pseudocode corresponding to (5) is listed below.
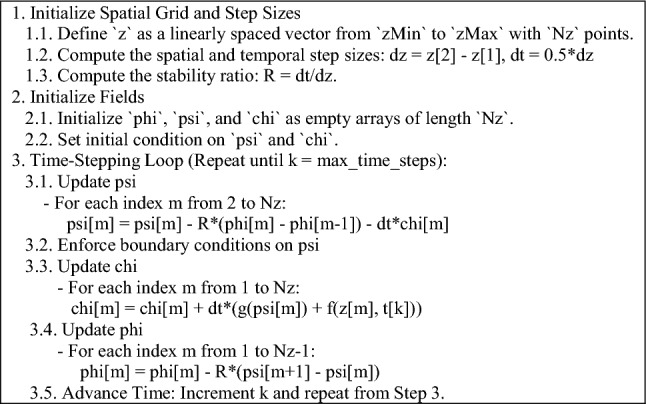


### Two-dimensional case

Let $\frac{\partial }{\partial z} = 0$ and ${{\varvec{\Psi}}}\left( {{\mathbf{r}},t} \right) = {\hat{\mathbf{z}}}\psi$ with ${\mathbf{r}} = {\hat{\mathbf{x}}}x + {\hat{\mathbf{y}}}y$. Then, ${{\varvec{\Phi}}}\left( {{\mathbf{r}},t} \right) = {\hat{\mathbf{x}}}\varphi_{x} + {\hat{\mathbf{y}}}\varphi_{y}$ and ${\mathbf{X}}_{\psi } \left( {{\mathbf{r}},t} \right) = {\hat{\mathbf{z}}}\chi$, leading to:

6 $$\left\{ {\begin{array}{*{20}l} {\frac{\partial }{\partial t}\varphi_{x} = - \frac{1}{\mu }\frac{\partial }{\partial y}\psi } \hfill \\ {\frac{\partial }{\partial t}\varphi_{y} = + \frac{1}{\mu }\frac{\partial }{\partial x}\psi } \hfill \\ {\frac{\partial }{\partial t}\psi = + \frac{1}{\varepsilon }\left( {\frac{\partial }{\partial x}\varphi_{y} - \frac{\partial }{\partial y}\varphi_{x} } \right) - \frac{1}{\varepsilon }\chi } \hfill \\ {\frac{\partial }{\partial t}\chi = + \frac{1}{\mu }\left[ {\beta \frac{\partial }{\partial t}\psi + wg\left( \psi \right) + f} \right]} \hfill \\ \end{array} } \right.$$

which is analog to the TE wave and can be called a $T\Psi$ wave. The pseudocode corresponding to (6) is listed below.


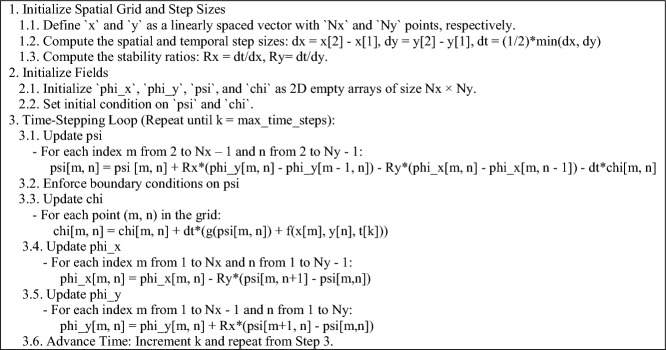


### Three-dimensional case

Let ${{\varvec{\Psi}}}\left( {{\mathbf{r}},t} \right) = {\hat{\mathbf{z}}}\psi$ with ${\mathbf{r}} = {\hat{\mathbf{x}}}x + {\hat{\mathbf{y}}}y + {\hat{\mathbf{z}}}z$. Then, ${{\varvec{\Phi}}}\left( {{\mathbf{r}},t} \right) = {\hat{\mathbf{x}}}\varphi_{x} + {\hat{\mathbf{y}}}\varphi_{y}$ and ${\mathbf{X}}_{\psi } \left( {{\mathbf{r}},t} \right) = {\hat{\mathbf{x}}}\chi_{x} + {\hat{\mathbf{y}}}\chi_{y} + {\hat{\mathbf{z}}}\chi_{z}$. Hence, (4) becomes:

7 $$\left\{ {\begin{array}{*{20}l} {\frac{\partial }{\partial t}\varphi_{x} = - \frac{1}{\mu }\frac{\partial }{\partial y}\psi } \hfill \\ {\frac{\partial }{\partial t}\varphi_{y} = + \frac{1}{\mu }\frac{\partial }{\partial x}\psi } \hfill \\ {\frac{\partial }{\partial t}\psi = + \frac{1}{\varepsilon }\left( {\frac{\partial }{\partial x}\varphi_{y} - \frac{\partial }{\partial y}\varphi_{x} } \right) - \frac{1}{\varepsilon }\chi_{z} } \hfill \\ {\chi_{x} = - \frac{\partial }{\partial z}\varphi_{y} } \hfill \\ {\chi_{y} = + \frac{\partial }{\partial z}\varphi_{x} } \hfill \\ {\frac{\partial }{\partial t}\chi_{z} = - \frac{1}{\mu }\frac{{\partial^{2} }}{{\partial z^{2} }}\psi + \frac{1}{\mu }\left[ {\beta \frac{\partial }{\partial t}\psi + wg\left( \psi \right) + f} \right]} \hfill \\ \end{array} } \right.$$

The pseudocode corresponding to (7) is listed subsequently.
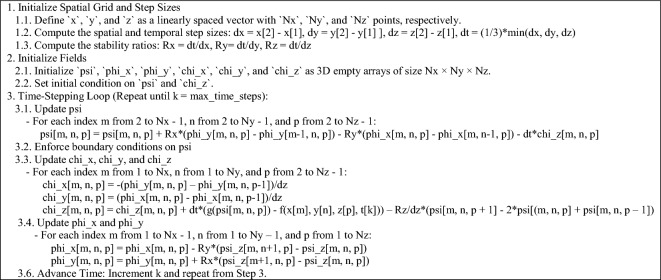


## Validation though numerical implementation

**Fig. 1:**
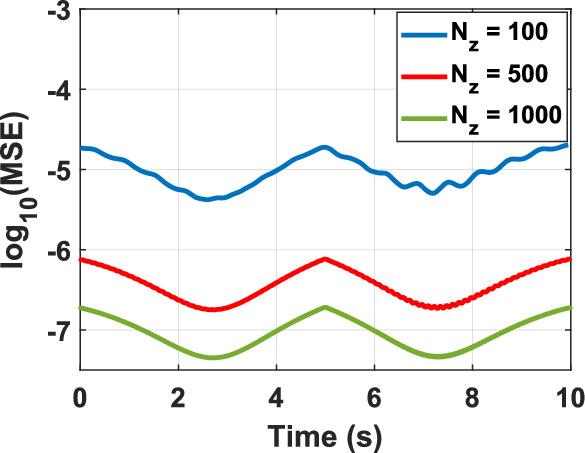
Convergence test for TP1.

**Fig. 2:**
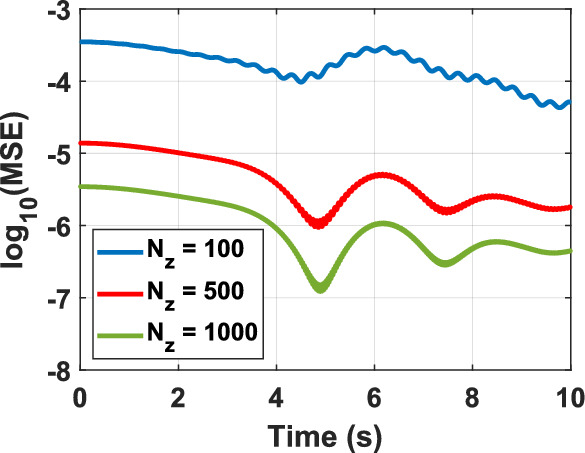
Convergence test for TP2.

**Fig. 3:**
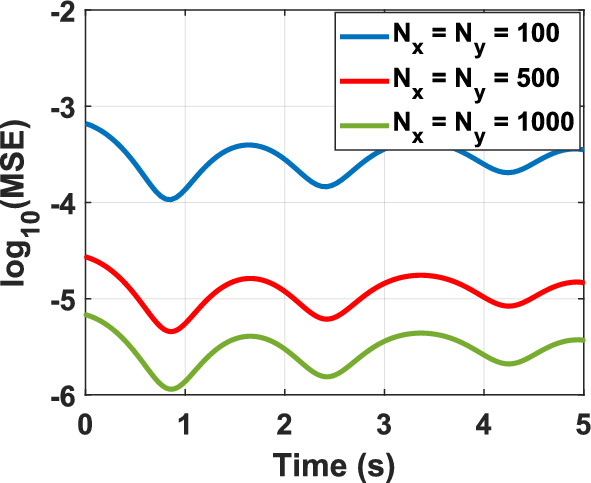
Convergence test for TP3.

**Fig. 4:**
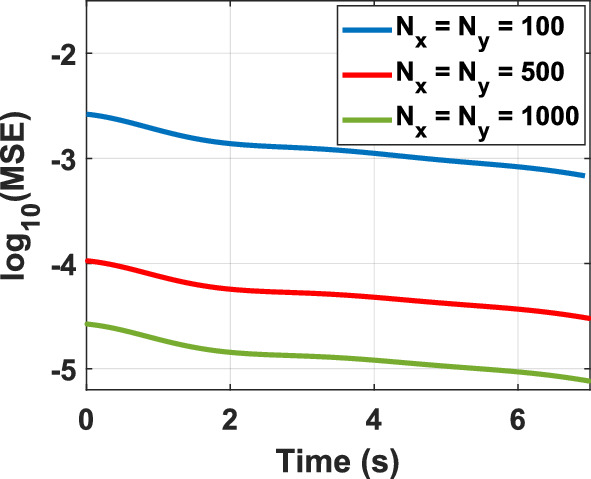
Convergence test for TP4.

**Fig. 5:**
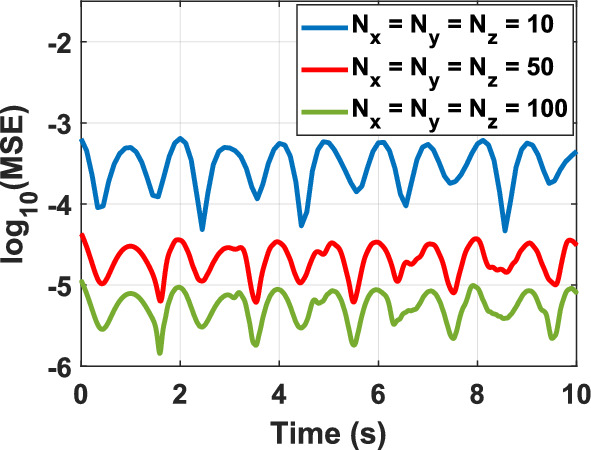
Convergence test for TP5 at $z \cong 1.5.$

**Fig. 6:**
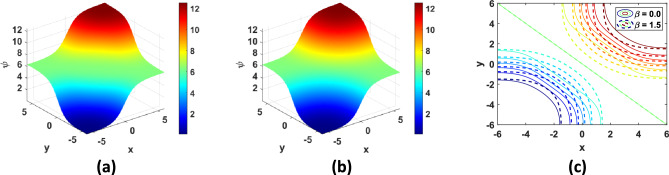
Solution of TP6 with $N = 100$ at $t = 3 {\text{s}}$: (**a**) 3D plot with $\beta = 0$, (**b**) 3D plot with $\beta = 1.5$, (**c**) contour plots.

**Fig. 7:**
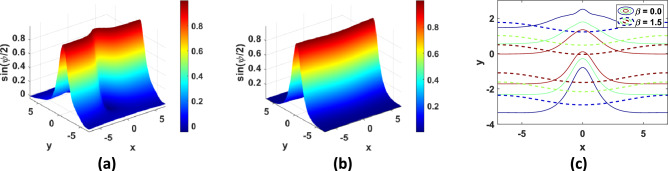
Solution of TP7 with $N = 100$ at $t = 7 {\text{s}}$: (**a**) 3D plot with $\beta = 0$, (**b**) 3D plot with $\beta = 1.5$, (**c**) contour plots.

**Fig. 8:**
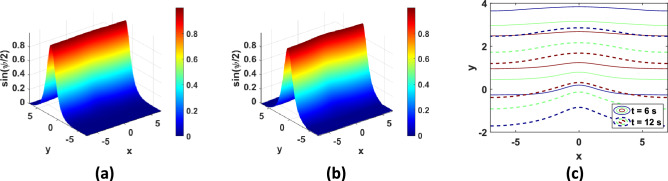
Solution of TP8 with $N = 100$: (**a**) 3D plot at $t = 6 {\text{s}}$, (**b**) 3D plot at $t = 12 {\text{s}}$, (**c**) contour plots.

**Fig. 9:**
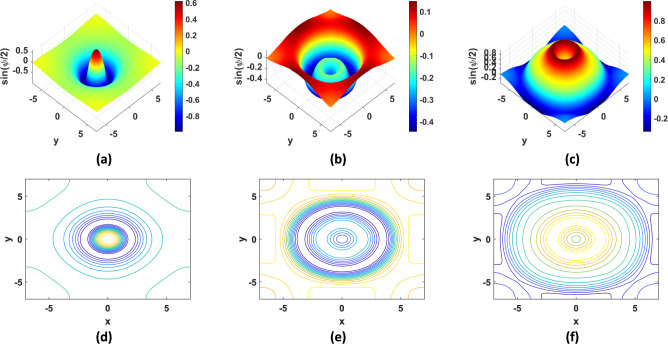
Solution of TP9 with $N = 100$ and $\beta = 0$: (**a**) 3D plot at $t = 5.6 {\text{s}}$, (**b**) 3D plot at $t = 8.4 {\text{s}}$, (**c**) 3D plot at $t = 11.2 {\text{s}}$, (**d**) contour plot at $t = 5.6 {\text{s}}$,( **e**) contour plot at $t = 8.4 {\text{s}}$, (**f**) contour plot at $t = 11.2 {\text{s}}$.

**Fig. 10:**
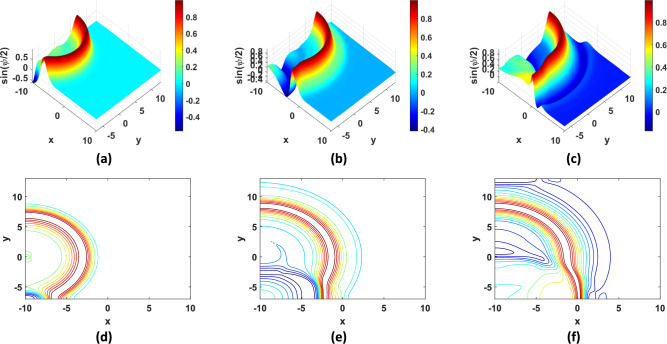
Solution of TP10 with $N = 100$ and $\beta = 0$: (**a**) 3D plot at $t = 4 {\text{s}}$, (**b**) 3D plot at $t = 8 {\text{s}}$, (**c**) 3D plot at $t = 11 {\text{s}}$, (**d**) contour plot at $t = 4 {\text{s}}$,(**e**) contour plot at $t = 8 {\text{s}}$, (**f**) contour plot at $t = 11 {\text{s}}$.

To assess the feasibility of the proposed representation of the KG equation from a computational perspective, various test problems (TPs) are numerically solved, as outlined in Table 1. The figure corresponding to the numerical solution of each problem matches the problem number, including figure 1-10. The boundary conditions (BCs) for the first five problems are Dirichlet, while the last five utilize homogeneous Neumann conditions. These correspond to the sine–Gordon equations, which include the collision of two circular ring solitons, a symmetrically perturbed static line soliton, a line soliton in a lossless inhomogeneous medium, a circular ring soliton, and the collision of two circular ring solitons^9^. In all problems, the weighting function is set to unity, except for test problem number eight, where $w = 1 + {\text{sech}}r$. For cases where the exact solution is available, all auxiliary conditions can be derived from the solution and are omitted for brevity. Additionally, validation is conducted through a convergence analysis. For problems without an exact solution, readers are referred to the relevant references to verify the correctness of the solutions. Following the Courant–Friedrichs–Lewy (CFL) condition, the time step-sizes are chosen as follows: $\frac{1}{2}\Delta z$ for 1D problems, $\frac{1}{2}\min \left\{ {\Delta x, \Delta y} \right\}$ for 2D, and $\frac{1}{3}\min \left\{ {\Delta x, \Delta y, \Delta z} \right\}$ for 3D problems. The number on nodes in the $x$, $y$, and $z$ dimensions are denoted by $N_{x}$, $N_{y}$, and $N_{z}$, respectively, and are set equal to $N$ in all dimensions for both 2D and 3D problems. For convergence studies, the mean squared error (MSE) is calculated using $MSE = \frac{1}{N}\mathop \sum \limits_{n = 1}^{N} \left[ {\psi_{ana} \left( {{\mathbf{r}}_{n} ,t} \right) - \psi_{num} \left( {{\mathbf{r}}_{n} ,t} \right)} \right]^{2}$, where $\psi_{ana}$ and $\psi_{num}$ represent the analytical and numerical solutions, respectively (Figs. 1, 2, 3, 4, 5, 6, 7, 8, 9 and 10).

**Table 1:** Test problems for validating the proposed FDTD scheme for the Klein–Gordon equation.

Test problem	Dimension	$g$	$f$	$\beta$	$\psi_{ana}$	References
1	1	$3/64$	0	0	$\sin \left( \frac{z}{8} \right)\left[ {\cos \left( \frac{t}{4} \right) - \sin \left( \frac{t}{4} \right)} \right]$	^12^
2	1	$\sin \left( \cdot \right)$	0	0	$4{\text{atan }}e^{{\left( {z - 0.5t} \right)/\sqrt {1 - 0.25} }}$	^40^
3	2	$\left( \cdot \right)^{3}$	$\left( {1 + \psi^{2} } \right)\psi$	0	$\cos x\cos y\sin t$	^41^
4	2	$\sin \left( \cdot \right)$	0	0	$4{\text{atan}}e^{x + y - t}$	^11^
5	3	$\left( \cdot \right)^{2}$	$\left( {2 + \psi } \right)\psi$	0	$\cos x\cos y\sin z\sin t$	^42^
6–-10	2	$\sin \left( \cdot \right)$	0	0, 1.5	NA	^9^

Numerical solutions for the first five TPs exhibit uniform convergence to the corresponding analytical solutions as the spatial grid is refined. In TP6 and TP7, an increase in the damping factor produces a progressive smoothing of the solution, which aligns with established physical intuition. TP9 and TP10 clearly display wave-like dynamics. Taken together, these results demonstrate that the proposed FDTD scheme is accurate, stable, and broadly applicable for the numerical solution of Klein–Gordon equations across a wide range of physical regimes.

## Representing the Klein–Gordon equation by symmetric Maxwellian fields

Similar to section two, the original form of the KG equation, given by

8 $$\left( {\frac{1}{{c^{2} }}\frac{{\partial^{2} }}{{\partial t^{2} }} - \nabla^{2} + \frac{{m^{2} c^{2} }}{{\hbar^{2} }}} \right)\psi \left( {{\mathbf{r}},t} \right) = 0$$

can be derived from Maxwellian vector fields. Specifically, consider the symmetric form of the MH equations that govern 3D complex-valued time-varying vector fields ${{\varvec{\Psi}}}$ and ${{\varvec{\Phi}}}$:

9 $$\left\{ {\begin{array}{*{20}l} {\nabla \times {{\varvec{\Psi}}} = - {\mathbf{X}}_{\varphi } - \mu \frac{\partial }{\partial t}{{\varvec{\Phi}}} } \hfill \\ {\nabla \times {{\varvec{\Phi}}} = + {\mathbf{X}}_{\psi } + \varepsilon \frac{\partial }{\partial t}{{\varvec{\Psi}}}} \hfill \\ {\nabla \cdot {{\varvec{\Psi}}} = \eta /\varepsilon \user2{ }} \hfill \\ {\nabla \cdot {{\varvec{\Phi}}} = \xi /\mu { }} \hfill \\ \end{array} } \right.$$

wherein one of the Cartesian components of ${{\varvec{\Psi}}}$ corresponds to the desired KG field variable. Unlike section two, divergences of the associated vector fields are also included, and the ${{\varvec{\Psi}}}$ field may have more than one non-zero component. An analogy between the MH and KG equations can be established if the source terms ${\mathbf{X}}_{\varphi }$ and ${\mathbf{X}}_{\psi }$ are selected such that:

10 $$\nabla \nabla \cdot {{\varvec{\Psi}}} + \nabla \times {\mathbf{X}}_{\varphi } + \mu \frac{\partial }{\partial t}{\mathbf{X}}_{\psi } \equiv \frac{{m^{2} c^{2} }}{{\hbar^{2} }}{{\varvec{\Psi}}}$$

As before, the terms ${\mathbf{X}}_{\varphi }$ and ${\mathbf{X}}_{\psi }$ can be understood as EM sources, and it is possible to define them in a way that satisfies Eq. (10). Additionally, all the theorems and concepts related to time-harmonic electromagnetic fields apply to the Maxwellian representation of the original Klein–Gordon (KG) equation^35^. Specifically, based on the equivalence theorem and the solutions to the Helmholtz equations, which include cylindrical and spherical harmonics, it can be concluded that for sources of finite extent, the decay rates of ${{\varvec{\Psi}}}$ and ${{\varvec{\Phi}}}$ is at least proportional to $r^{ - 1}$ and $r^{ - 2}$ in two and three dimensions. Furthermore, in addition to ${{\varvec{\Phi}}}$, which serves as the dual vector field of ${{\varvec{\Psi}}}$, the proposed representation of the KG equation introduces new elements including:

11 $$\left\{ {\begin{array}{*{20}l} {\nabla \cdot {\mathbf{X}}_{\psi } + \frac{\partial }{\partial t}\eta = 0\;\left( {{\text{continuity equation regarding }}{\mathbf{X}}_{\psi } } \right)} \hfill \\ {\nabla \cdot {\mathbf{X}}_{\varphi } + \frac{\partial }{\partial t}\xi = 0\;\left( {{\text{continuity equation regarding }}{\mathbf{X}}_{\varphi } } \right) } \hfill \\ {{{\varvec{\Lambda}}} = {\text{Re}}\left\{ {{{\varvec{\Psi}}} \times {{\varvec{\Phi}}}^{\user2{*}} } \right\}\;\left( {\text{Poynting vector}} \right)} \hfill \\ {u = \frac{1}{2}\varepsilon \left| {{\varvec{\Psi}}} \right|^{2} + \frac{1}{2}\mu \left| {{\varvec{\Phi}}} \right|^{2} \;\left( {\text{stored energy density}} \right) } \hfill \\ {\frac{ - \partial }{{\partial t}}u = \nabla \cdot {{\varvec{\Lambda}}} + {\text{Re}}\left\{ \Delta \right\} \;\left( {\text{Poynting theorem}} \right) } \hfill \\ \end{array} } \right.$$

wherein ${\Delta } = {{\varvec{\Psi}}} \cdot {\mathbf{X}}_{\psi }^{*} + {{\varvec{\Phi}}}^{*} \cdot {\mathbf{X}}_{\varphi }$. Thus, if the source terms are such that ${\text{Re}}\left\{ \Delta \right\}$ vanishes, the Poynting theorem can serve as a desirable conservation law. Specifically, the non-negative scalar field $u$ may be a better choice than the conventional probability current density of the KG field.

The central issue which should be address is the existence of source terms that ensure the condition ${\text{Re}}\left\{ \Delta \right\} = 0$, which will be referred to as the *Fundamental Conservation Relation* (FCR) from now on. Furthermore, Maxwellian vector fields that satisfy this condition will be called *Conserved Maxwellian Fields* (CMF). The sufficient condition to ensure FCR is that $\Delta = 0$, which is a simpler to work with. It is important to note that one can relate the source terms using the expression ${\mathbf{X}}_{\varphi } = - \frac{{{{\varvec{\Psi}}} \cdot {\mathbf{X}}_{\psi }^{*} }}{{{{\varvec{\Phi}}}^{\user2{*}} \cdot {{\varvec{\Phi}}}^{\user2{*}} }}{{\varvec{\Phi}}}^{\user2{*}}$ to construct CMFs. However, this choice may lead to singular solution due to zero crossings of ${{\varvec{\Phi}}}$. Additionally, this method may not be feasible if the primary interest lies in the plane-wave solution, as the resulting solution space may not be complete.

## Plane-wave solution of the Maxwellian Klein–Gordon equation

The most common solution type used to ensure completeness of the functional space is the plane-wave solution, which, for the original KG equation—i.e., (8)—takes the form:

12 $$\psi \left( {{\mathbf{r}},t} \right) = \mathop \sum \limits_{p} \left\{ {A_{p} e^{{ - j\left( {\omega_{p} t - {\mathbf{k}}_{p} \cdot {\mathbf{r}}} \right)}} + C_{p} e^{{ + j\left( {\omega_{p} t - {\mathbf{k}}_{p} \cdot {\mathbf{r}}} \right)}} } \right\}$$

with the dispersion relation given by $k_{p}^{2} = \left( {\omega_{p} /c} \right)^{2} - \left( {mc/\hbar } \right)^{2}$. Here, $j$ denotes the imaginary unit, $\hbar$ is the reduced Planck’s constant and $m$ is the particle mass^32^. The focus of this work will be on determining the coefficients $A_{p}$ such that the FCR holds. The coefficients $C_{p}$ can be obtained in a similar manner. It is worth noting that any field component beyond the scalar KG solution may represent a potential hidden aspect of QFT. Let the vector fields ${{\varvec{\Psi}}}$ and ${{\varvec{\Phi}}}$ be defined as:

13 $$\left\{ {\begin{array}{*{20}c} {\Psi = \mathop \sum \limits_{p} \left( {{\hat{\mathbf{x}}}A_{px} + {\hat{\mathbf{y}}}A_{py} + {\hat{\mathbf{z}}}A_{pz} } \right)\psi_{p} } \\ {\Phi = \mathop \sum \limits_{p} \left( {{\hat{\mathbf{x}}}B_{px} + {\hat{\mathbf{y}}}B_{py} + {\hat{\mathbf{z}}}B_{pz} } \right)\psi_{p} } \\ \end{array} } \right.$$

where $\psi_{p} = e^{{ - j\left( {\omega_{p} t - {\mathbf{k}}_{p} \cdot {\mathbf{r}}} \right)}}$. By the linearity of (9), such expansions are valid. Furthermore, in view of the dispersion relation, (10) is automatically satisfied for this basis. Assume that the $z$ component of ${{\varvec{\Psi}}}$ corresponds to the scalar KG field of interest. This requires $A_{pz} \ne 0$ for at least one $p$. Additionally, let $A_{px} = \alpha_{px} A_{pz}$, $A_{py} = \alpha_{py} A_{pz}$, $B_{px} = \beta_{px} A_{pz}$, $B_{py} = \beta_{py} A_{pz}$, and $B_{pz} = \beta_{pz} A_{pz}$. The goal is to determine $\alpha_{px}$, $\alpha_{py}$, $\beta_{px}$, $\beta_{py}$, and $\beta_{pz}$ such that (13) satisfies the curl equations from (9), together with $\Delta = 0$. This, in turn, determines $\nabla \cdot {{\varvec{\Psi}}}$ and $\nabla \cdot {{\varvec{\Phi}}}$. It can be shown that $\Delta = 0$ is equivalent to

14 $$\begin{gathered} \gamma_{qx}^{*} \left( { - \alpha_{py} k_{qz} + k_{qy} + \gamma_{px} + \alpha_{py} k_{pz} - k_{py} } \right) + \gamma_{qy}^{*} \left( {\alpha_{px} k_{qz} + k_{qx} + \gamma_{py} + k_{px} - \alpha_{px} k_{pz} } \right) \hfill \\ + \gamma_{qz}^{*} \left( { - \alpha_{px} k_{qy} + \alpha_{py} k_{qx} + \gamma_{pz} + \alpha_{px} k_{py} - \alpha_{py} k_{px} } \right) = \left[ {k_{qx}^{2} + k_{qy}^{2} + k_{qz}^{2} + \left( {mc/\hbar } \right)^{2} } \right] \hfill \\ \left( {1 + \alpha_{px} \alpha_{qx}^{*} + \alpha_{py} \alpha_{qy}^{*} } \right) \hfill \\ \end{gathered}$$

wherein $\gamma_{pv} = \mu \omega_{p} \beta_{pv} , v = x,y,z$. Clearly, (14) is a highly nonlinear equation, and thus, it would be informative to go through its solution step-by-step; from one to three dimensions, to construct the corresponding CMFs.

### CMF in one dimension

Let $k_{px} = k_{pz} = 0$ and $k_{py} \ne 0$. Then, (14) simplifies to:

15 $$\gamma_{qx}^{*} \left( {k_{qy} + \gamma_{px} - k_{py} } \right) + \gamma_{qy}^{*} \gamma_{py} - \alpha_{px} \gamma_{qz}^{*} \left( {k_{qy} - \gamma_{pz} /\alpha_{px} - k_{py} } \right) = \left[ {k_{qy}^{2} + \left( {mc/\hbar } \right)^{2} } \right]\left( {1 + \alpha_{px} \alpha_{qx}^{*} + \alpha_{py} \alpha_{qy}^{*} } \right)$$

Since the right-hand side contains a constant term, let $\gamma_{px} - k_{py} = c_{x}$ and $\gamma_{pz} /\alpha_{px} - k_{py} = c_{z}$, where $c_{x}$ and $c_{z}$ are complex constants. Substituting these into (15), it can be verified that it holds provided $\gamma_{py} = \alpha_{py} = 0$ and $c_{x} = \pm c_{z} = \pm jmc/\hbar$ . Accordingly, for a given set of $\alpha_{px}$,

16 $$\left\{ {\begin{array}{*{20}l} {\Psi = \mathop \sum \limits_{p} \left( {{\hat{\mathbf{x}}}\alpha_{px} + {\hat{\mathbf{z}}}} \right)A_{pz} \psi_{p} } \hfill \\ {\Phi = \mathop \sum \limits_{p} \left[ {{\hat{\mathbf{x}}}\left( {k_{py} \pm jmc/\hbar } \right) - {\hat{\mathbf{z}}}\left( {k_{py} \pm jmc/\hbar } \right)\alpha_{px} } \right]\frac{1}{{\mu \omega_{p} }}A_{pz} \psi_{p} } \hfill \\ \end{array} } \right.$$

This solution can be interpreted as an elliptically polarized $T\Psi \Phi$ quantum wave, propagating along the positive $y$-axis. Notably, since the coefficients $\alpha_{px}$ are arbitrary, there exists an infinite family of 1D solutions. However, if only the original KG scalar solution is of interest, one may choose $\alpha_{px} = 0$, resulting in a linearly polarized wave and reducing the solution space to two. Thus, the scalar field taken as the $z$ component of ${{\varvec{\Psi}}}$, induces a corresponding single-component ${{\varvec{\Phi}}}$ such that the pair $\left( {{{\varvec{\Psi}}},{{\varvec{\Phi}}}} \right)$ form a 1D CMF. The fields are related via

17 $${{\varvec{\Phi}}}_{p} = \frac{{{\mathbf{k}}_{p} \pm jmc/\hbar }}{{\mu \omega_{p} }} \times {{\varvec{\Psi}}}_{p}$$

wherein the subscript $p$ denotes the corresponding vector component. The aforesaid time-domain solution resembles TEM wave propagation in lossy media in the time-harmonic regime, with ${\mathbf{k}}_{p} \pm jmc/\hbar$ acting as a complex wave vector. Nevertheless, despite the difference in the solution domain, the fields are non-decaying propagating fields. Interestingly, the phase difference between the vector fields is proportional to the particle mass and vanishes for a massless particle, similar to the behavior of a TEM wave in unbounded, lossless media. Moreover, the ratio between transverse field components is $\frac{{\omega_{p} \mu }}{{k_{py} \pm jmc/\hbar }}$ , which duo to the dispersion relation, leads to

18 $$\left| {\psi_{px} /\varphi_{pz} } \right| = \left| {\psi_{pz} /\varphi_{px} } \right| = \sqrt {\mu /\varepsilon }$$

and mimics the wave impedance of a classical TEM mode in free space. Finally, the resulting CMF is symmetric, satisfying $\nabla \cdot {{\varvec{\Psi}}} = \nabla \cdot {{\varvec{\Phi}}} = 0$. An interested reader may verify the existence of an alternative 1D solution to Eq. (13) by assuming $k_{py} = k_{pz} = 0$ and $k_{px} \ne 0$. However, no solution exists for the case $k_{px} = k_{py} = 0$ and $k_{pz} \ne 0$.

### CMF in two dimensions

Let $k_{pz} = 0$ and $k_{px} k_{py} \ne 0$, which simplifies (14) to:

19 $$\begin{gathered} \gamma_{qx}^{*} \left( {k_{qy} + \gamma_{px} - k_{py} } \right) + \gamma_{qy}^{*} \left( { - k_{qx} + \gamma_{py} + k_{px} } \right) + \gamma_{qz}^{*} \left( { - \alpha_{px} k_{qy} + \alpha_{py} k_{qx} + \gamma_{pz} + \alpha_{px} k_{py} - \alpha_{py} k_{px} } \right) \hfill \\ = \left[ {k_{qx}^{2} + k_{qy}^{2} + \left( {mc/\hbar } \right)^{2} } \right]\left( {1 + \alpha_{px} \alpha_{qx}^{*} + \alpha_{py} \alpha_{qy}^{*} } \right) \hfill \\ \end{gathered}$$

Proceeding as in the 1D case, let $\gamma_{px} - k_{py} = c_{y}$ and $\gamma_{py} + k_{px} = c_{x}$, where $c_{x}$ and $c_{y}$ are complex constants. Then, (19) holds if $\gamma_{pz} = \alpha_{px} = \alpha_{py} = 0$, $c_{x} = j\sigma_{x}$ and $c_{y} = j\sigma_{y}$ with $\left( {\sigma_{x} ,\sigma_{y} } \right) \in {\mathbb{R}}^{2}$ satisfying $\sigma_{x}^{2} + \sigma_{y}^{2} = \left( {mc/\hbar } \right)^{2}$. Consequently,

20 $$\left\{ {\begin{array}{*{20}l} {\Psi = \mathop \sum \limits_{p} {\hat{\mathbf{z}}}A_{pz} \psi_{p} } \hfill \\ {\Phi = \mathop \sum \limits_{p} \left[ {{\hat{\mathbf{x}}}\left( {k_{py} + j\sigma_{y} } \right) - {\hat{\mathbf{y}}}\left( {k_{px} - j\sigma_{x} } \right)} \right]\frac{1}{{\mu \omega_{p} }}A_{pz} \psi_{p} } \hfill \\ \end{array} } \right.$$

This solution can be interpreted as a linearly polarized $T\Psi$ quantum wave, propagating along the positive $z$-axis. As in the 1D case, the number of 2D solutions is infinite, due to the arbitrary choice of $\left( {\sigma_{x} ,\sigma_{y} } \right)$ on the circle defined by the mass constraint. Thus, the scalar KG field, represented by the $z -$ components of ${{\varvec{\Psi}}}$, induces a two-component field ${{\varvec{\Phi}}}$, yielding a 2D CMF. The relationship between the fields can be compactly written as

21 $${{\varvec{\Phi}}}_{p} = \frac{{{\mathbf{k}}_{p} \pm j{{\varvec{\upsigma}}}}}{{\mu \omega_{p} }} \times {{\varvec{\Psi}}}_{p}$$

wherein ${{\varvec{\upsigma}}} = {\hat{\mathbf{x}}}\sigma_{x} + {\hat{\mathbf{y}}}\sigma_{y}$. Additionally, the impedance-like magnitude ratio satisfies:

22 $$\left| {\psi_{pz} /\varphi_{px} } \right|^{2} + \left| {\psi_{pz} /\varphi_{py} } \right|^{2} = \mu /\varepsilon$$

Thus, both the magnitude and phase of the dual field ${{\varvec{\Phi}}}$ are controlled by the position on the circle defined by $\left( {\sigma_{x} ,\sigma_{y} } \right)$. However, unlike the 1D case, the resulting CMF is not symmetric, because

23 $$\left\{ {\begin{array}{*{20}l} {\nabla \cdot \Psi = 0} \hfill \\ {\nabla \cdot \Phi = - \mathop \sum \limits_{p} \frac{{{ }\sigma_{y} k_{px} + \sigma_{x} k_{py} }}{{\mu \omega_{p} }}A_{pz} \psi_{p} \ne 0} \hfill \\ \end{array} } \right.$$

Finally, one may verify that no other 2D solution exists for (16).

### CMF in three dimensions

A rather tedious algebraic analysis shows that no 3D CMF solution exists if $\alpha_{px} = \alpha_{py} = 0$ or if both $\alpha_{px} \ne 0$ and $\alpha_{py} \ne 0$. However, following the same strategy as in lower dimensions, two nontrivial 3D solutions are possible under asymmetric conditions.

*Case* 1. $$\alpha_{px} = 0$$ and $$\alpha_{py} \ne 0$$.

Assume the auxiliary field relations $\gamma_{px} + \alpha_{py} k_{pz} - k_{py} = j\sigma_{x}$, $\gamma_{py} + k_{px} = j\sigma_{y}$, and $\gamma_{pz} - \alpha_{py} k_{px} = j\sigma_{z}$, wherein $\left( {\sigma_{x} ,\sigma_{y} ,\sigma_{z} } \right) \in {\mathbb{R}}^{3}$ satisfies $\sigma_{x}^{2} + \sigma_{y}^{2} + \sigma_{z}^{2} = \left( {mc/\hbar } \right)^{2}$. Substituting into (14), one finds a valid solution if

24 $$\left\{ {\begin{array}{*{20}l} {\sigma_{x} = mc/\hbar } \hfill \\ {\sigma_{y} = \sigma_{z} = 0} \hfill \\ {\alpha_{py} = \frac{{ - k_{pz} }}{{k_{py} - jmc/\hbar }}} \hfill \\ \end{array} } \right.$$

The resulting fields are:

25 $$\left\{ {\begin{array}{*{20}l} {{{\varvec{\Psi}}}^{\left( 1 \right)} = \mathop \sum \limits_{p} \left( { - {\hat{\mathbf{y}}}\frac{{k_{pz} }}{{k_{py} - jmc/\hbar }} + {\hat{\mathbf{z}}}} \right)A_{pz} \psi_{p} } \hfill \\ {{{\varvec{\Phi}}}^{\left( 1 \right)} = \mathop \sum \limits_{p} \left[ {{\hat{\mathbf{x}}}\frac{{\left( {\omega_{p} /c} \right)^{2} - k_{px}^{2} }}{{k_{py} - jmc/\hbar }} - {\hat{\mathbf{y}}}k_{px} - {\hat{\mathbf{z}}}\frac{{k_{px} k_{pz} }}{{k_{py} - jmc/\hbar }}} \right]\frac{1}{{\mu \omega_{p} }}A_{pz} \psi_{p} } \hfill \\ \end{array} } \right.$$

This solution introduces a fully 3D ${{\varvec{\Phi}}}^{\left( 1 \right)}$, and also adds a *y*-component to ${{\varvec{\Psi}}}^{\left( 1 \right)}$. Moreover,

26 $${{\varvec{\Phi}}}_{p}^{\left( 1 \right)} = \frac{{{\mathbf{k}}_{p} \pm {\hat{\mathbf{y}}}\left( {jmc/\hbar } \right)}}{{\mu \omega_{p} }} \times {{\varvec{\Psi}}}_{p}^{\left( 1 \right)}$$

and

27 $$\left\{ {\begin{array}{*{20}l} {\nabla \cdot {{\varvec{\Psi}}}^{\left( 1 \right)} = \left( {mc/\hbar } \right)\mathop \sum \limits_{p} \frac{{{ }k_{pz} }}{{k_{py} - jmc/\hbar }}A_{pz} \psi_{p} \ne 0} \hfill \\ {\nabla \cdot {{\varvec{\Phi}}}^{\left( 1 \right)} = - \frac{{\left( {mc/\hbar } \right)}}{\mu }\mathop \sum \limits_{p} \frac{{{ }k_{px} }}{{\omega_{p} }}A_{pz} \psi_{p} \ne 0 } \hfill \\ \end{array} } \right.$$

*Case* 2. $$\alpha_{px} \ne 0$$ and $$\alpha_{py} = 0$$.

Let $\gamma_{px} - k_{py} = j\sigma_{x}$, $\gamma_{py} + k_{px} - \alpha_{px} k_{pz} = j\sigma_{y}$, and $\gamma_{pz} + \alpha_{px} k_{py} = j\sigma_{z}$ with the same constrain on $\left( {\sigma_{x} ,\sigma_{y} ,\sigma_{z} } \right)$ as before. Then, (14) admits a solution for:

28 $$\left\{ {\begin{array}{*{20}l} {\sigma_{x} = \sigma_{z} = 0} \hfill \\ {\sigma_{y} = mc/\hbar } \hfill \\ {\alpha_{px} = \frac{{ - k_{pz} }}{{k_{px} + jmc/\hbar }}} \hfill \\ \end{array} } \right.$$

The fields then become:

29 $$\left\{ {\begin{array}{*{20}l} {{{\varvec{\Psi}}}^{\left( 2 \right)} = \mathop \sum \limits_{p} \left( { - {\hat{\mathbf{x}}}\frac{{k_{pz} }}{{k_{px} + jmc/\hbar }} + {\hat{\mathbf{z}}}} \right)A_{pz} \psi_{p} \user2{ }} \hfill \\ {{{\varvec{\Phi}}}^{\left( 2 \right)} = \mathop \sum \limits_{p} \left[ {{\hat{\mathbf{x}}}k_{py} - {\hat{\mathbf{y}}}\frac{{\left( {\omega_{p} /c} \right)^{2} - k_{py}^{2} }}{{k_{px} + jmc/\hbar }} + {\hat{\mathbf{z}}}\frac{{k_{py} k_{pz} }}{{k_{px} + jmc/\hbar }}} \right]\frac{1}{{\mu \omega_{p} }}A_{pz} \psi_{p} } \hfill \\ \end{array} } \right.$$

This structure mirrors that of the previous case, leading to:

30 $${{\varvec{\Phi}}}_{p}^{\left( 2 \right)} = \frac{{{\mathbf{k}}_{p} \pm {\hat{\mathbf{x}}}\left( {jmc/\hbar } \right)}}{{\mu \omega_{p} }} \times {{\varvec{\Psi}}}_{p}^{\left( 2 \right)}$$

And

31 $$\left\{ {\begin{array}{*{20}l} {\nabla \cdot {{\varvec{\Psi}}}^{\left( 2 \right)} = - \left( {mc/\hbar } \right)\mathop \sum \limits_{p} \frac{{{ }k_{pz} }}{{k_{px} + jmc/\hbar }}A_{pz} \psi_{p} \ne 0} \hfill \\ {\nabla \cdot {{\varvec{\Phi}}}^{\left( 2 \right)} = - \frac{{\left( {mc/\hbar } \right)}}{\mu }\mathop \sum \limits_{p} \frac{{{ }k_{py} }}{{\omega_{p} }}A_{pz} \psi_{p} \ne 0} \hfill \\ \end{array} } \right.$$

Thus, in contrast to 1D and 2D cases, each with infinite number of solutions, there are only two 3D solutions. Furthermore, similar to the 1D case, the resulting CMF is symmetric. Specifically, both of the vector fields of a 3D CMF have monopole sources. Another intriguing aspect of these solutions is their formal analogy with the plane‑wave spinors of the Dirac equation^43^. In the special case $k_{px} = k_{py} = 0$, one may identify the propagation direction with the spin quantization along the z-axis. Under this reduction (25) and (29) leads to:

32 $$\left\{ {\begin{array}{*{20}l} {{{\varvec{\Psi}}}_{p}^{\left( 1 \right)} = \left( {{\hat{\mathbf{y}}}\frac{{k_{pz} }}{jmc/\hbar } + {\hat{\mathbf{z}}}} \right)A_{pz} \psi_{p} } \hfill \\ {{{\varvec{\Phi}}}_{p}^{\left( 1 \right)} = - {\hat{\mathbf{x}}}\frac{{\left( {\omega_{p} /c} \right)^{2} }}{jmc/\hbar }\frac{1}{{\mu \omega_{p} }}A_{pz} \psi_{p} } \hfill \\ \end{array} } \right.$$

and

33 $$\left\{ {\begin{array}{*{20}l} {{{\varvec{\Psi}}}_{p}^{\left( 2 \right)} = \left( { - {\hat{\mathbf{x}}}\frac{{k_{pz} }}{jmc/\hbar } + {\hat{\mathbf{z}}}} \right)A_{pz} \psi_{p} } \hfill \\ {{{\varvec{\Phi}}}_{p}^{\left( 2 \right)} = - {\hat{\mathbf{y}}}\frac{{\left( {\omega_{p} /c} \right)^{2} }}{jmc/\hbar }\frac{1}{{\mu \omega_{p} }}A_{pz} \psi_{p} } \hfill \\ \end{array} } \right.$$

Equations (32) and (33) exhibit a striking structural resemblance to the spin-up and spin-down solutions of the Dirac equation, particularly in their orthogonality and component organization. Like the mutually orthogonal, these solutions form an orthogonal set. Furthermore, each “spinor” in the proposed framework consists of a single-component dual field ${{\varvec{\Phi}}}$ coupled with a two-component primary field ${{\varvec{\Psi}}}$, mirroring the way Dirac spinors for opposite spin projections distribute their non-zero momentum components along the quantization axis. This parallelism extends to antiparticles: the CMF solutions associated with the $C_{p}$​ coefficients exhibit a charge-conjugation-like duality, akin to the $v\left( p \right)$ spinors in the Dirac theory.

## Discussion

As demonstrated, the Maxwellian reformulation of the KG equation not only offers an efficient computational framework for numerical analysis but also reveals deeper theoretical implications for QFT. Central to this reformulation is the emergence of a dialectical structure found in classical electromagnetism: the electric and magnetic fields mutually regenerate one another through temporal and spatial coupling. The embedded nature of Lenz’s law, which ensures that induced effects oppose their causes, further reinforces this interpretation of structural opposition. This dialectical character contrasts with the structure of traditional quantum equations such as the Schrödinger, KG, and Dirac equations, each of which involves only a single field variable and thus lacks internal dynamical interplay or “dialogue”^44^. In contrast, the Maxwellian framework comprises interacting field pairs that exhibit mutual causality. Viewed through a dialectical lens^45,46^, one may interpret the electric field as a “thesis”, the magnetic field as its “antithesis”, and the energy flux described by the Poynting vector as the resulting “synthesis”. This self-regenerating, bidirectional structure presents a richer field dynamic that scalar formulations inherently lack.

This reformulation also invites reflection on the role of symmetry in physics and aesthetics. While symmetry is often considered a hallmark of beauty in both natural systems and physical theory^47^, in practice, perfect symmetry is rarely—if ever—realized in macroscopic nature. Instead, partial or broken symmetry dominates, as observed in biological structures, crystal lattices, and cosmological distributions. Even in gauge theories and field models, spontaneous symmetry breaking is often essential to physical realism^48,49^. From this perspective, one may argue that dialectical balance more accurately reflects the elegance of nature than strict symmetry. Therefore, in the search for fundamental physical laws, it may be fruitful to focus on frameworks with interacting elements, such as those found in the Maxwellian field system, rather than isolated, symmetric scalar fields. Furthermore, it is well known that Dirac’s primary motivation in formulating his equation was to construct a relativistically consistent wave equation that is first-order in both space and time^50^—a property already realized in Maxwell’s equations. The symmetry exhibited in 3D CMFs introduced in this work is likewise partial: although the fields evolve in a structured and coupled manner, their divergence expressions are not identical, indicating incomplete symmetry. Rather than being a flaw, this asymmetry aligns with how nature operates.

Finally, the proposed analogy between plane-wave solutions of Dirac fields and the 3D CMFs, while conceptually intriguing, is not entirely unexpected. Both systems are governed by first-order differential equations in space and time and are associated with conservation laws of identical mathematical form. This resemblance reinforces the interpretive link between the proposed Maxwellian reformulation of the KG equation and the deeper structure of field theory.

## Conclusion

This work demonstrates that the KG equation can be reformulated as a first-order system analogous to MH equations, enabling its solution via a Yee-based FDTD method. The numerical scheme developed is validated across a range of linear and nonlinear test problems in one, two, and three dimensions, showing stability, accuracy, and versatility in complex media. Beyond numerical efficiency, the reformulation introduces the concept of CMFs, which satisfy a non-negative conservation law derived from plane-wave quantization. This provides a meaningful alternative to the problematic probability density interpretation of the KG field. In higher dimensions, especially in 3D, CMFs exhibit structural symmetry and divergence consistent with monopole-like sources, a feature absent from the original scalar formulation. Notably, the 3D CMF solutions exhibit formal analogies with Dirac spinors in both structure and conservation properties. This suggests a potential reinterpretation of scalar field equations in terms of more fundamental vector field dynamics and raises the possibility of previously unrecognized degrees of freedom. The proposed framework not only bridges classical electromagnetics with relativistic field theory but also opens new directions in numerical quantum field modeling and theoretical exploration.

## Data Availability

Data sets generated during the current study are available from the corresponding author on reasonable request.

## Code availability

The simulation codes used in this study are available from the corresponding author upon request.
